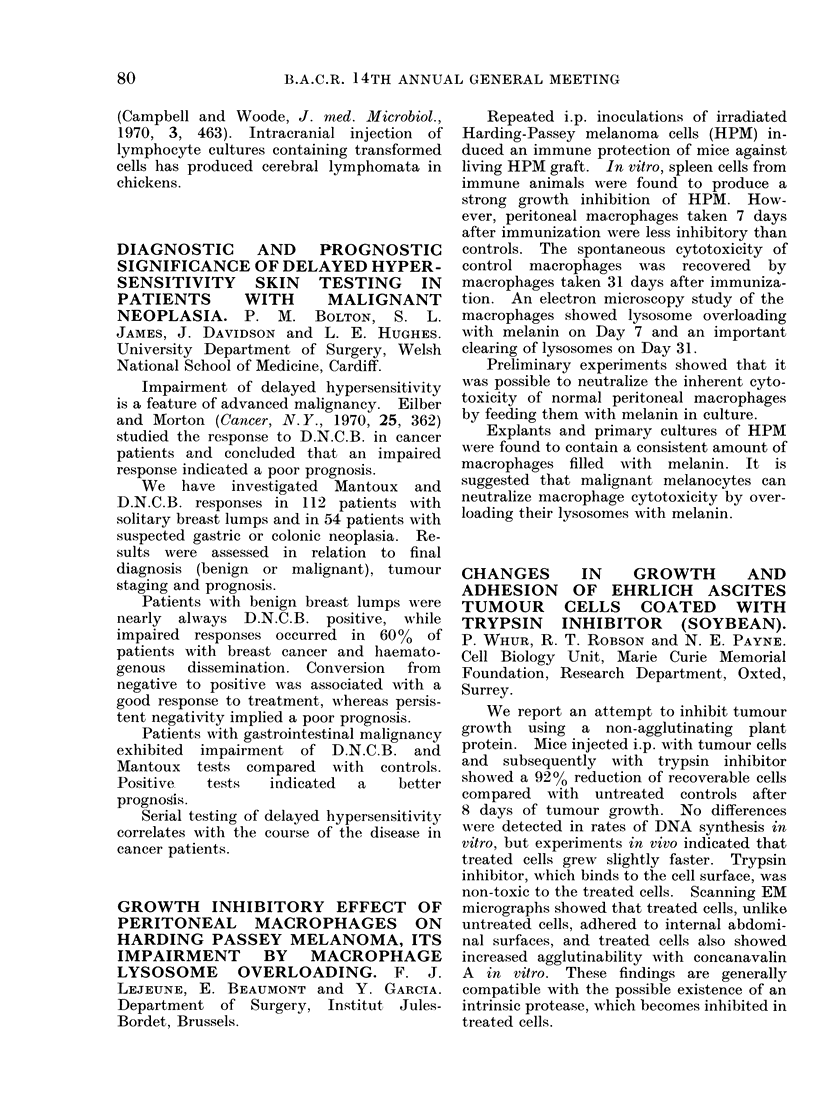# Growth inhibitory effect of peritoneal macrophages on Harding Passey melanoma, its impairment by macrophage lysosome overloading.

**DOI:** 10.1038/bjc.1973.87

**Published:** 1973-07

**Authors:** F. J. Lejeune, E. Beaumont, Y. Garcia


**GROWTH INHIBITORY EFFECT OF PERITONEAL MACROPHAGES ON HARDING PASSEY MELANOMA, ITS IMPAIRMENT BY MACROPHAGE LYSOSOME OVERLOADING.** F. J. Lejeune, E. Beaumont and Y. Garcia. Department of Surgery, Institut Jules-Bordet, Brussels.

Repeated i.p. inoculations of irradiated Harding-Passey melanoma cells (HPM) induced an immune protection of mice against living HPM graft. *In vitro*, spleen cells from immune animals were found to produce a strong growth inhibition of HPM. However, peritoneal macrophages taken 7 days after immunization were less inhibitory than controls. The spontaneous cytotoxicity of control macrophages was recovered by macrophages taken 31 days after immunization. An electron microscopy study of the macrophages showed lysosome overloading with melanin on Day 7 and an important clearing of lysosomes on Day 31.

Preliminary experiments showed that it was possible to neutralize the inherent cytotoxicity of normal peritoneal macrophages by feeding them with melanin in culture.

Explants and primary cultures of HPM were found to contain a consistent amount of macrophages filled with melanin. It is suggested that malignant melanocytes can neutralize macrophage cytotoxicity by overloading their lysosomes with melanin.